# Three-dimensional mapping of ultrasound-derived skeletal muscle shear wave velocity

**DOI:** 10.3389/fbioe.2023.1330301

**Published:** 2023-12-21

**Authors:** Tobias Götschi, Jess G. Snedeker, Daniel P. Fitze, Fabio Sarto, Jörg Spörri, Martino V. Franchi

**Affiliations:** ^1^ Orthopaedic Biomechanics Laboratory, Department of Orthopaedics, Balgrist University Hospital, Zurich, Switzerland; ^2^ Institute for Biomechanics, ETH Zurich, Zurich, Switzerland; ^3^ Department of Orthopaedics, Sports Medical Research Group, Balgrist University Hospital, University of Zurich, Zurich, Switzerland; ^4^ Department of Orthopaedics, University Centre for Prevention and Sports Medicine, Balgrist University Hospital, University of Zurich, Zurich, Switzerland; ^5^ Department of Biomedical Sciences, Institute of Physiology, University of Padua, Padua, Italy

**Keywords:** shear wave elastography, muscle, biomechanics, ultrasound, stiffness, elasticity

## Abstract

**Introduction:** The mechanical properties of skeletal muscle are indicative of its capacity to perform physical work, state of disease, or risk of injury. Ultrasound shear wave elastography conducts a quantitative analysis of a tissue’s shear stiffness, but current implementations only provide two-dimensional measurements with limited spatial extent. We propose and assess a framework to overcome this inherent limitation by acquiring numerous and contiguous measurements while tracking the probe position to create a volumetric scan of the muscle. This volume reconstruction is then mapped into a parameterized representation in reference to geometric and anatomical properties of the muscle. Such an approach allows to quantify regional differences in muscle stiffness to be identified across the entire muscle volume assessed, which could be linked to functional implications.

**Methods:** We performed shear wave elastography measurements on the vastus lateralis (VL) and the biceps femoris long head (BFlh) muscle of 16 healthy volunteers. We assessed test-retest reliability, explored the potential of the proposed framework in aggregating measurements of multiple subjects, and studied the acute effects of muscular contraction on the regional shear wave velocity post-measured at rest.

**Results:** The proposed approach yielded moderate to good reliability (ICC between 0.578 and 0.801). Aggregation of multiple subject measurements revealed considerable but consistent regional variations in shear wave velocity. As a result of muscle contraction, the shear wave velocity was elevated in various regions of the muscle; showing pre-to-post regional differences for the radial assessement of VL and longitudinally for BFlh. Post-contraction shear wave velocity was associated with maximum eccentric hamstring strength produced during six Nordic hamstring exercise repetitions.

**Discussion and Conclusion:** The presented approach provides reliable, spatially resolved representations of skeletal muscle shear wave velocity and is capable of detecting changes in three-dimensional shear wave velocity patterns, such as those induced by muscle contraction. The observed systematic inter-subject variations in shear wave velocity throughout skeletal muscle additionally underline the necessity of accurate spatial referencing of measurements. Short high-effort exercise bouts increase muscle shear wave velocity. Further studies should investigate the potential of shear wave elastography in predicting the muscle’s capacity to perform work.

## Introduction

The mechanical properties of muscles are of relevance in the context of clinical examination and various scientific endeavors. During voluntary contraction, a muscle’s stiffness can be related to muscle functional properties, as it is directly related to the tension it produces (Ettema and Huijing, 1994; Morgan, 1977), while localized alterations of stiffness may underlie deleterious conditions, including dysfunctional innervation, muscle contractures and fibrosis (Kawai et al., 2018; Alfuraih et al., 2019). Assessing the change in passive muscle stiffness over the corresponding joint’s range of motion yields an estimate for passive tension (Johns and Wright, 1962; Gennisson et al., 2010; Miyamoto et al., 2018; Wang et al., 2019), which in turn may be pivotal in understanding certain injury mechanisms or may help explain conditions of idiopathic musculoskeletal pain or dysfunction (Vandervoort, 1999). Muscle stiffness at rest depends on its structure and composition as well as the nature of any preceding stimuli (Siracusa et al., 2019). Moreover, the extracellular matrix (ECM), the intramuscular connective tissue network of skeletal muscle, is considered a key element contributing to whole muscle stiffness (Kjær, 2004; Fouré et al., 2011). For instance, repeated high-effort muscular contraction generates ECM creep, potentially disturbing the finely tuned interplay between the contractile and noncontractile elements, which may in part account for peripheral fatigue (Siracusa et al., 2019; Lacourpaille et al., 2017).

Whereas manual palpation provides a simple and useful means to assess muscle stiffness (Kvåle et al., 2003), more sophisticated approaches are needed to quantify this muscle feature. Shear wave elastography (SWE) has arisen as one method of choice because it yields quantitative estimates of tissue mechanical properties. Localized displacement induces shear motion propagating through the tissue, the velocity of which is in part dependent on tissue stiffness, with increasing stiffness yielding increasing shear wave velocity (SWV) (Nightingale, 2011). In principle, any soft tissue imaging modality with sufficient spatial and temporal resolution can be used to observe shear wave propagation, but ultrasound (US) has specific appeal due to its low cost and large availability. Moreover, the US transducer can induce the required tissue micromotion by transmitting properly timed compressive waves that superimpose into localized shear displacement.

US SWE has been successfully used on skeletal muscle to estimate active and passive mechanical tension (Hug et al., 2015; Zimmer et al., 2022), to detect disease (Alfuraih et al., 2019; Boulard et al., 2021; Tisha et al., 2018) or exercise-induced damage (Siracusa et al., 2019; Lacourpaille et al., 2017; Chalchat et al., 2020; Lacourpaille et al., 2014) and other stimuli (Sions et al., 2012). The most commonly used US SWE systems work with one-dimensional piezo array transducers, which consequently yield quasi-two-dimensional (2D) measurements of tissue stiffness. 2D arrays of piezo elements for three-dimensional (3D) SWE exist, but their volume of view is limited (Dong et al., 2022).

For the assessment of large structures such as skeletal muscle, 2D US SWE hence carries a significant limitation in that a single measurement only samples a minute portion of the volume of interest. However, the rate of measurement of state-of-the-art US devices is sufficiently high (∼2 Hz) that even large skeletal muscles can be sampled in their entirety at a relatively high spatial resolution within a few minutes. Provided that each measurement is annotated with its respective position and the movement of the structure of interest during the scan is negligible or accounted for, the set of acquired measurements can be projected into 3D space to yield a volumetric SWV representation of the structure (Götschi et al., 2021). To enable intra- and inter-individual comparisons of the local SWV, the retrieved volume can be mapped into an abstracted representation of the muscle of study in reference to selected geometrical and anatomical features. A similar approach has already been shown to be technically feasible, reproducible and clinically significant in previous studies for tendons (Götschi et al., 2021; Götschi et al., 2022a; Götschi et al., 2022b). However, it is not clear *a priori* whether this is directly transferable to muscle tissue, particularly given the much larger volumes of interest.

In the current proof-of-concept study, we propose and assess a framework for spatially resolved, three-dimensional anatomically referenced skeletal muscle US SWE measurements. Specifically, we aimed to (1) determine the test-retest reliability of the proposed approach, (2) explore its potential in aggregating measurements of multiple subjects, and (3) assess its capability of detecting changes in the three-dimensional shear wave velocity patterns, such as those induced by muscle contraction.

## Materials and methods

### Study design and study population

In the current study, we performed SWE measurements on the right vastus lateralis (VL) muscle and the left biceps femoris long head (BFlh) muscle of 16 adult participants who reported being free of any lower extremity musculoskeletal injuries/complaints. (eight females; age: 27.3 ± 2.8 years; height: 174.4 ± 9.2 cm; weight: 67.3 ± 9.2 kg; BMI: 22.1 ± 2.4 kg m^-2^).

For both muscles, first, two US SWE measurements were performed to determine the reliability of the method; then, a maximum effort task was performed specifically targeting both muscles, consisting of one isometric knee extension for VL and six repetitions of Nordic Hamstring Exercise (NHE) for BFlh muscle, immediately followed by a third US SWE measurement. Additionally, the BFlh muscle was scanned again 5 minutes after the initial post-contraction measurement. The maximum eccentric hamstring strength (MEHS) performed during the NHE was used to quantify the physical performance during the BFlh contraction exercise and explore any potential associations with the muscle’s SWV. The study involving humans was approved by the Cantonal Ethics Committee Zurich, Switzerland (KEK-ZH-NR: 2017-01395). All participants were informed in writing about the measurement procedures and provided written consent.

### Shear wave elastography measurement

Participants laid on a physiotherapy bed at least 5 minutes prior to the first acquisition to allow body fluid stabilization and minimize potential confounding factors related to preceding physical activity. For both muscles, the portion between 0% and 70% of the femur length (where 0 was regarded as the mid-patellar point) was measured. The proximal measurement border was determined based on the distance between the patella center and the greater trochanter and marked with a permanent ink pen (Franchi et al., 2020a). For the VL measurements, the participants laid supine on the examination table. For the BFlh measurements, the participants laid prone on the examination table with their feet just outside the table frame. The initial measurements of both muscles were repeated once by the same operator between which the participants lied onto their back and then re-established the measurement position. Immediately after the respective muscle contraction tasks described below, we performed another SWE measurement. The BFlh was scanned 5 min after the first post-contraction measurement once more to track acute changes in SWV over a short period of time.

### Maximum effort excercise

The maximum effort exercise for the VL muscle consisted of a 15-s isometric contraction of the knee extensors. The participants sat on the examination table with the knees flexed 90° and the right ankle fixated by a brace. For the BFlh contraction exercise, the participants performed 6 maximum eccentric knee flexion manoeuvres on a hamstring exercise board (NordBord, Vald Performance, Newstead, Australia). The participants were positioned with their knees on a padded board, without shoes, with the ankles secured by braces just above the l ateral malleoli. They were advised to maintain alignment of their shoulders, hips, and knees while crossing their arms in front of their body. They were then instructed to slowly advance forward and exert maximum resistance against the movement using both legs (Kiers et al., 2021). During exercise execution, we recorded the maximum force achieved by the participant during each repetition using the force sensors integrated into the exercise board (Opar et al., 2013). The six recorded maximum force values were aggregated by extracting the median, which was then normalized by the participant’s body weight to derive the normalized MEHS.

### Shear wave elastography acquisition

The data acquisition procedure has been technically validated and described in detail previously (Götschi et al., 2021). Briefly, measurements of shear wave group velocity were acquired as provided by the ultrasound device (Aixplorer Ultimate, SuperSonic Imagine, Aix-en-Provence, France) using a linear 5 cm transducer (SuperLinear SL18-5). These measurements were transmitted on the fly to the measurement computer via ethernet and the MATLAB interface provided by the manufacturer. The transducer pose was tracked throughout the scan with an optical tracking system and optical markers attached to the transducer. We acquired both brightness-mode (B-mode) and SWV measurements in parallel. The B-mode images were reconstructed analogously to the SWV measurements, provided the basis for anatomical orientation and were segmented manually to mask the respective SWV volume. During scanning, the transducer was oriented parallel to the muscle fibres, and was carefully kept constantly in plane throughout the whole ROI by an expert operator (MVF). Adequate orientation can be ensured by observing and maximizing the striped appearance of the intramuscular structure in relation to transducer rotation (about its long axis). The region of interest was scanned in multiple consecutive swipes with the starting location randomized (distal/proximal). SWV measurements were acquired at 2 Hz.

### Parameterization of the shear wave velocity map

**FIGURE 1:**
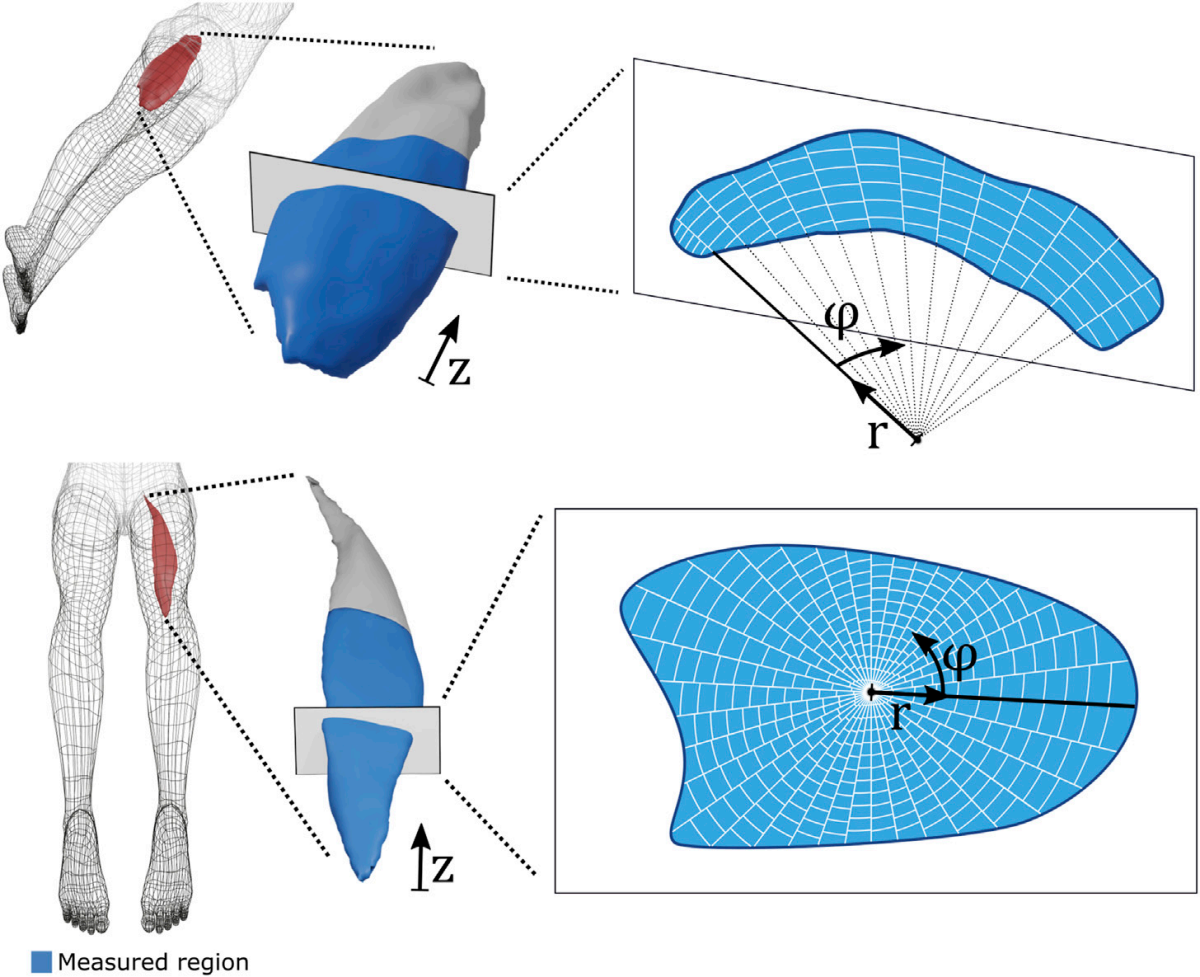
Spatial parameterization of volumetric shear wave velocity (SWV) maps. Top row: Vastus lateralis SWV volumes were discretized into longitudinal slices (along z), and each slice was subdivided into angular (φ) and radial (r) partitions in reference to the centre of a best-fit circle (Taubin, 1991). Bottom row: Biceps femoris long head SWV volumes were handled analogously with the sole difference that the origin of the polar coordinate system was placed at the geometric mean of the respective cross-sectional slice.

To conduct meaningful spatially resolved comparisons and aggregations between different SWE acquisitions, each measurement was transformed into a standardized form. This was achieved by mapping the gridded SWV volume into a geometrically and anatomically referenced representation. Each VL measurement was first aligned along its distal-proximal axis (using principal component analysis). Slices orthogonal to the distal-proximal axis were then retrieved (eight and six for the VL and the BFlh, respectively), and each slice was mapped into a polar coordinate system with its origin given by the circle segment that best fit the respective segmentation mask (least squares). All shear wave velocity estimates within a slice were aggregated into discrete cells of uniform relative radial and azimuthal extent. BFlh measurements were processed analogously with the sole difference that the slice-specific polar coordinate system’s origin was given by the geometric mean of the respective segmentation mask. Figure 1 visualizes the parameterization procedure for the VL (top row) and the BFlh (bottom row).

### Statistical analysis

Descriptive statistics are presented as the mean and standard deviation. Test-retest reliability was quantified in terms of the intraclass correlation coefficient (ICC(2,1)) (Shrout and Fleiss, 1979) based on a two-way random effects model assessing the absolute agreement of a single-measure approach and the related standard error of measurement (Sem) (de Vet et al., 2006). ICC values were classified as poor (≤0.2), fair (0.21–0.4), moderate (0.41–0.6), good (0.61–0.8), and very good (>0.8) (Ashby, 1991). We reported both the reliability of whole-muscle measurements and the reliability of measuring a distinct region of the muscle. The latter measurement was evaluated in both the context of assessing attributes within a subject-muscle (inter-regional) or across subjects (inter-subject). Whole muscle reliability metrics were reported with their estimates and the associated 95% confidence intervals. Analysis of regional reliability requires aggregation of different metrics; hence, we reported median and interquartile range. We conducted paired-sampled t-tests to evaluate the effect of muscular contraction on muscle SWV across both global and regional measurements stratified along one of three dimensions. To explore associations between normalized MEHS and BFlh SWV, we applied Spearman rank correlation tests at both the global and regional levels. The analysis was conducted with MATLAB (2022b, The MathWorks, Inc., Natick, MA, USA). Statistical significance was set at α = 0.05.

## Results

### Global muscle assessment

Whole-muscle measurements were highly reliable in both assessed structures (Table 1).

**TABLE 1:** Test-retest reliability of whole-muscle SWV measurements. ICC: Intra class correlation coefficient. CI: Confidence interval. SEm: Standard error of measurement.

Structure	ICC (95% CI)	SEm [m/s]
Vastus lateralis	0.941 (0.843, 0.979)	0.044
Biceps femoris long head	0.885 (0.709, 0.958)	0.058

**FIGURE 2:**
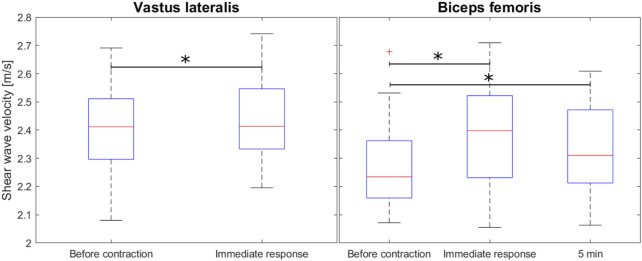
Overall shear wave velocity before and after voluntary muscular contraction for the VL (15 s of isometric maximal effort) and after 6 repetions of the Nordic Hamstring Exercise for the BFlh muscle).

The VL resting SWV was lower than the BFlh SWV (*p* = 0.025). Immediately following muscular contraction, SWV was elevated in both structures (VL: *p* = 0.026, BFlh: *p* = 0.002), and BFlh SWV remained elevated at the third measurement 5 min after the contraction (*p* = 0.038) (Figure 2).

Whereas normalized MEHS was non-significantly associated with initial BFlh SWV (Spearman correlation coefficient: ρ = −0.485, *p* = 0.059) or absolute change in BFlh SWV (*ρ* = −0.076, *p* = 0.780), it was significantly associated with the immediate post-contraction BFlh SWV (*ρ* = −0.588, *p* = 0.019).

### Regional muscle assessment

Regional muscle assessments yielded moderate to very good reliability. Inter-regional assessments yielded higher reliability than inter-subject assessments (Table 2).

**TABLE 2:** Median test-retest reliability of regional muscle SWV measurements with regards to a repeated assessment of muscle regions within the same subject (Inter-regional) or over multiple subjects (Inter-subject). IQR: Interquartile range. SEm: Standard error of measurement.

Structure	Domain	ICC (IQR)	SEm (IQR) [m/s]
Vastus lateralis	Inter-regional	0.752 (0.673, 0.815)	0.328 (0.289, 0.396)
	Inter-subject	0.600 (0.406, 0.727)	0.273 (0.185, 0.409)
Biceps femoris long head	Inter-regional	0.801 (0.566, 0.843)	0.223 (0.190, 0.252)
	Inter-subject	0.578 (0.419, 0.714)	0.227 (0.170, 0.303)

**FIGURE 3:**
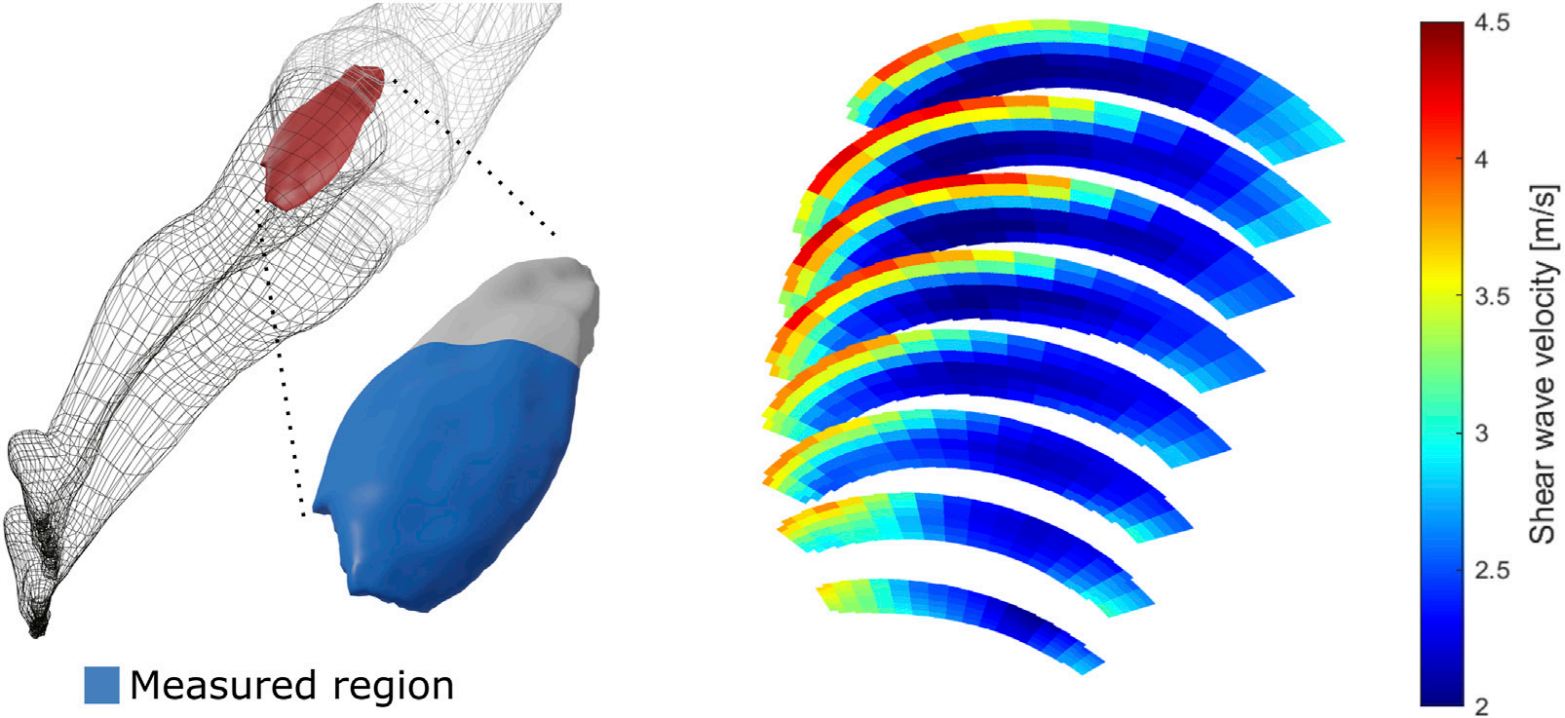
Average vastus lateralis regional shear wave velocity at rest. The left side of the figure presents a schematic depiction of the vastus lateralis muscle with the measured region in blue. The right side of the figure shows the three-dimensional measurements of shear wave velocity of the same muscle at rest, averaged over all participant measurements.

**FIGURE 4:**
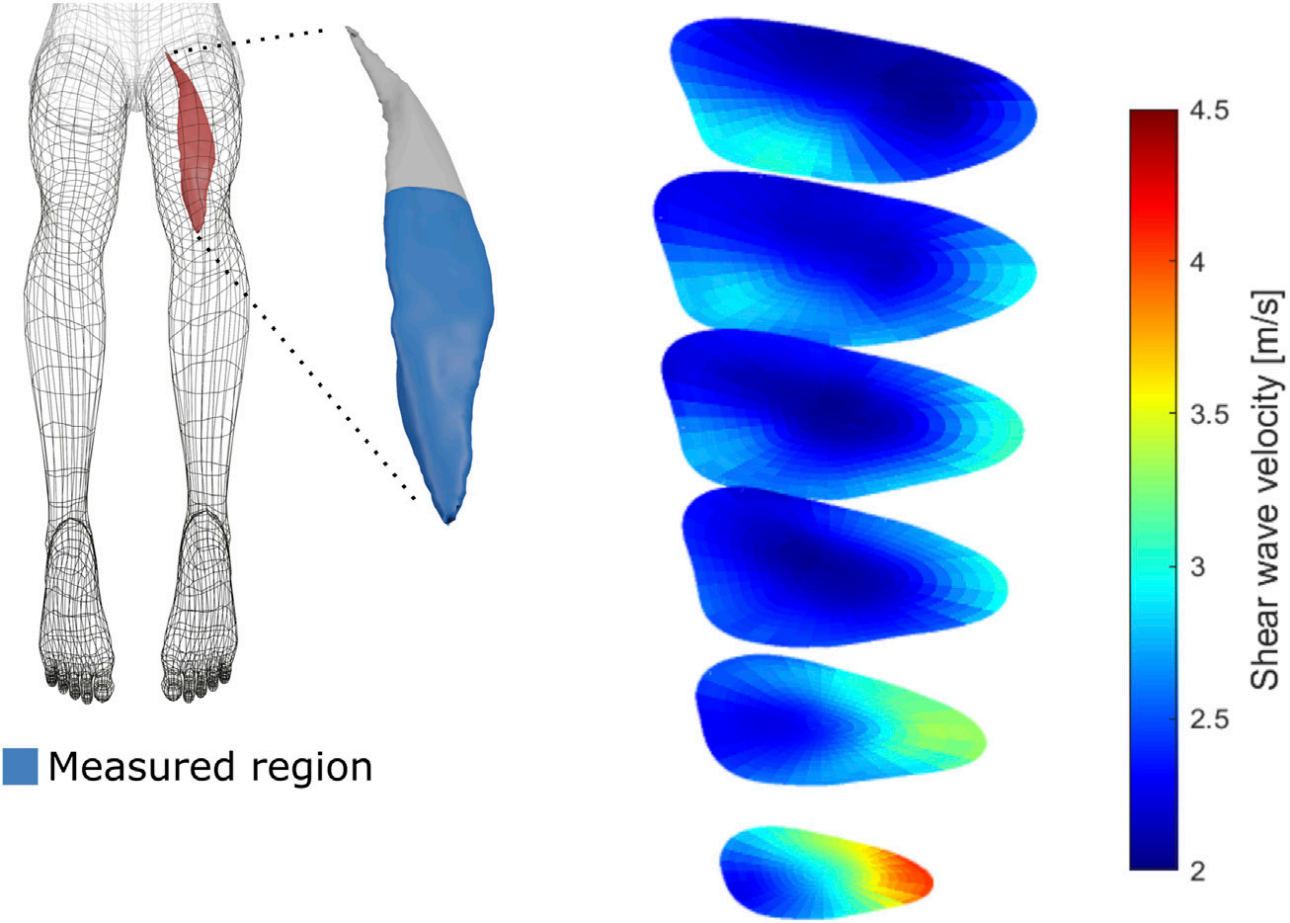
Average biceps femoris long head regional shear wave velocity at rest. The left side of the figure presents a schematic depiction of the biceps femoris long head muscle with the measured region in blue. The right side of the figure shows the three-dimensional measurements of shear wave velocity of the same muscle at rest, averaged over all participant measurements.

Aggregation of multiple subject measurements revealed considerable but consistent regional variations in shear wave velocity. Figure 3 and Figure 4 provide visualizations of the parameterized SWV maps of the VL and the BFlh, respectively averaged over the pre-exercise measurements of all subjects.

**FIGURE 5:**
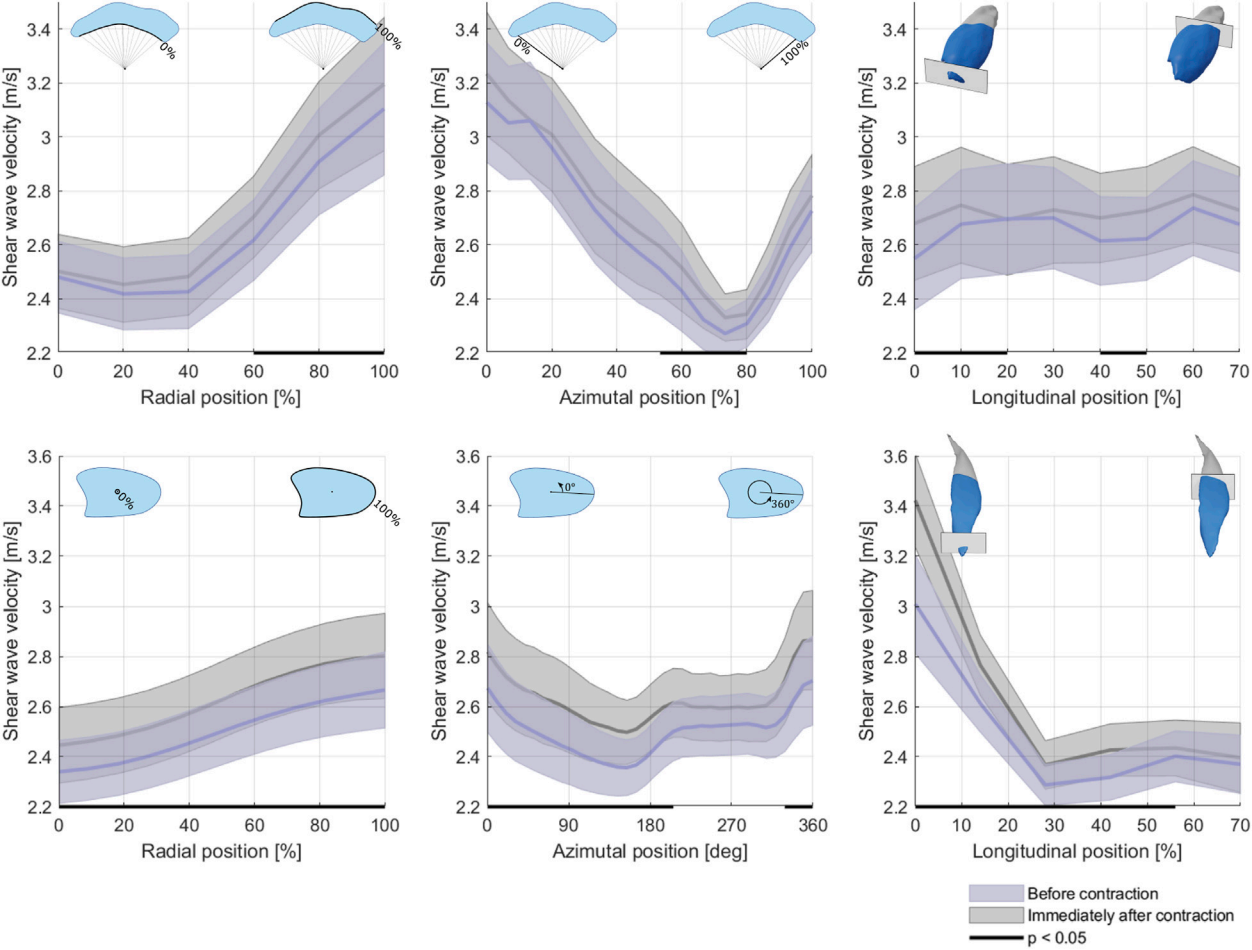
Average shear wave velocity over the radial (first column), angular (second column) and longitudinal axes of the vastus lateralis (top row) and biceps femoris long head (bottom row) at rest before and immediately after contraction. Area of uncertainty: Standard error of the mean.

The VL SWV showed a strong gradient over the radial axis of the muscle, with deep regions displaying lower SWV than superficial regions. Over the angular axis, a U-shaped relationship was evident, with central regions possessing lower SWV compared to the periphery (Figure 5, top row). The BFlh SWV displayed a steady increase from the radial centre to the muscle surface. The distal portion displayed considerably higher SVW than the central and proximal regions (Figure 5, bottom row).

Preceding muscular contraction generally elevated SWV; however, no strong indicator for a region-specific response was evident.

**FIGURE 6:**
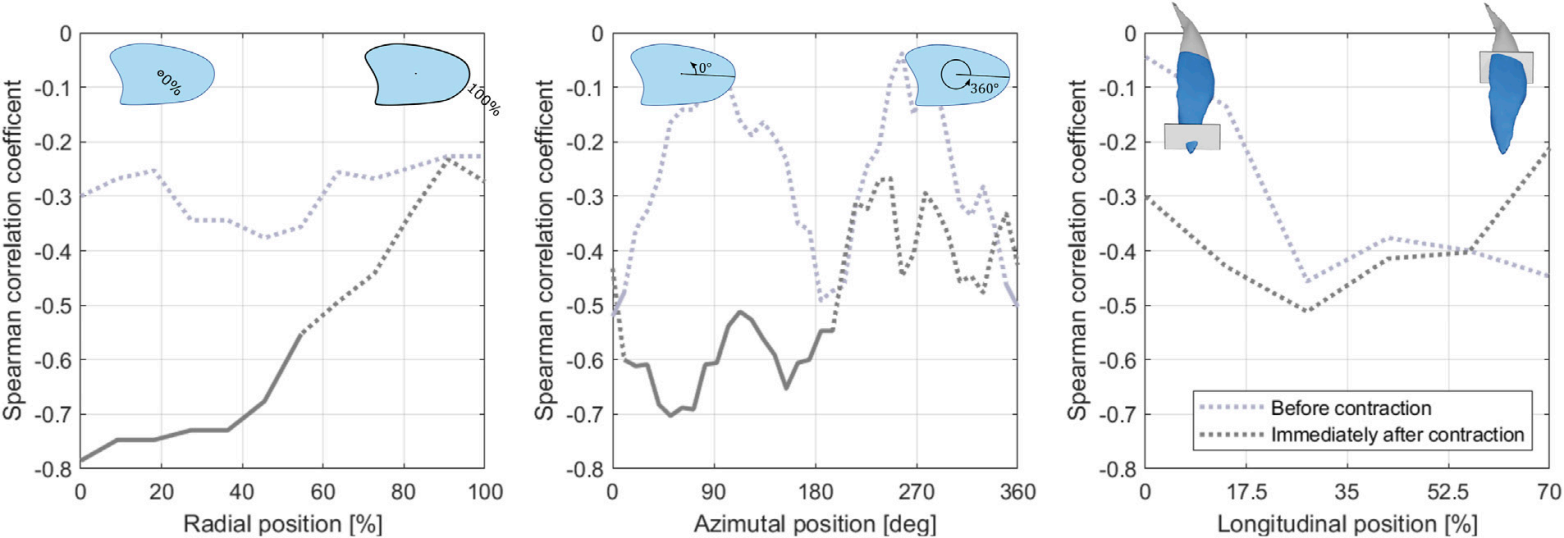
Association of the normalized maximum eccentric hamstring strength and the regional (from left to right: radial, angular, longitudinal) biceps femoris long head shear wave velocity before (blue line) and immediately after the exercise bout (grey line). Regions with associations of statistical significance (*p* < 0.05) are delineated with a solid line.

We did, however, find strong regionality in the association between normalized MEHS and BFlh SWV. Specifically, normalized MEHS correlated strongly with the SWV assigned to the central portion (in the radial axis) of the muscle immediately after the exercise bout but not with the one at rest (Figure 6).

## Discussion

### Test-retest reliability of global and regional muscle assessments

The presented approach revealed reliable results in both muscles assessed. Indeed, global muscle assessment reliability compares favorably with the available literature where reported ICC values for 2D SWV assessments are in the range of 0.800—0.937 (Lacourpaille et al., 2012; Phan et al., 2019; Bravo-Sánchez et al., 2021) and 0.842—0.850 (Lee et al., 2021; Šarabon et al., 2019) for the VL and the BFlh, respectively. VL measurements yielded higher reliability than BFlh measurements possibly because of the more complex architecture of the latter (Pimenta et al., 2018; Franchi et al., 2020b; Brusco et al., 2022), which usually presents a characteristic “s-like shape” fascicle architecture requiring careful alignment of the US transducer during the measurement (Charles et al., 2022). The vastus lateralis muscle, although showing regional architectural differences, is known to present a more “homogeneous” architecture compared to other muscle groups (Blazevich et al., 2006; Franchi et al., 2018; Sarto et al., 2021). Furthermore, the more irregular shape of BFlh complicated its segmentation on the US reconstruction which may have induced additional measurement variability. Global muscle assessments were also more reliable than regional assessments in both muscles. This indicates that many factors modulating muscle shear properties act on the global muscle or subject scale. Spatially resolved measurements suffer from random variability introduced by registration inaccuracies and other noise-generating processes that outweigh systematic regional variation. Many of these inaccuracies are dominant on the between-subject level, and hence, interregional within-subject reliability was superior to inter-subject reliability.

In both muscles, the aggregation of all subject measurements revealed large but consistent variations in SWV, underlining the necessity of accurate spatial referencing of the measurements.

### Three-dimensional shear wave velocity variations across different anatomical locations

Our novel approach unveiled, for the first time, distinct regional differences in stiffness within human muscles. Specifically, the deeper regions of the VL displayed lower SWV than superficial regions, while the BFlh exhibited increases from the radial center to the muscle surface. Notably, the VL exhibited a U-shaped relationship over the angular axis, with central regions displaying lower stiffness compared to the periphery (i.e., medial and lateral regions of the VL). Additionally, distal portions of the BFlh showed remarkably higher SWV compared to more proximal regions. These location-dependent muscle mechanical properties may significantly contribute to providing new insights into muscle force production and susceptibility to muscle strain injuries in different regions. Several factors could account for these anatomical differences in muscle stiffness observed in different muscle regions. No previous studies have investigated potential regional differences in ECM distribution in humans. However, animal studies have shown that ECM sheaths at the muscle periphery are directly connected to the epimysium, the external layer of dense connective tissue which ensheaths the entire muscle (Sleboda et al., 2020). As the epimysium is known to possess larger collagen fibres, the ECM of external muscle regions may present similar properties, partially explaining the higher SWV at the muscle periphery observed in our study.

In addition, depth-dependent differences in fibre phenotype have been previously documented in seminal cadaver studies (Lexell et al., 1983a; Lexell et al., 1983b), with higher type I fibre percentages reported in deeper compartments of the VL. As resting tension is known to be higher in fast than in slow fibres (Schiaffino and Reggiani, 2011):this may contribute to the lower stiffness observed in deep regions of the VL, although this is just a speculation. Furthermore, the differences in motor unit potential properties observed when comparing different VL depths (Knight and Kamen, 2005; Jones et al., 2021) might suggest a differential muscle innervation profile, which could influence muscle tone. Last, regional differences in intramuscular fat content can also play a role, as it is generally considered inversely associated with muscle stiffness (Pinel et al., 2021). In support of this view, the region where we observed the lower stiffness in BFlh (∼40% of femur length) is known to have the highest amount of intramuscular fat in the hamstring muscles (Yoshiko et al., 2017). The determinants of anatomical differences in muscle stiffness warrant further investigation in future studies.

### Muscle contraction-induced changes in the three-dimensional shear wave velocity patterns

We observed a spatially uniform increase in SVW in response to both a 15-s isometric contraction exercise for the VL and six eccentric contractions for the BFlh. There are multiple reports in the literature that investigate the 2D shear wave velocity of skeletal muscle at rest in response to physical work. Siracusa et al. had their subjects perform 60 repetitions of 5 s isometric maximum voluntary knee extensions and measured the SWV in one location in the VL (∼50% longitudinal, central in the mediolateral axis, in the superficial half of the muscle). SWV was measured every 10 repetitions and showed a significant decrease after the first 10 repetitions followed by further depression, reaching a minimum after 50 repetitions (Siracusa et al., 2019). In accordance with Siracusa et al.’s findings, an isometric trunk extension fatigue protocol depressed the apparent shear modulus of the deep multifidus muscles (Vatovec et al., 2022). Similarly, low-intensity, high-duration loading as generated during a long-distance race elicited a depression in SWV that persisted for at least 72 h (Andonian et al., 2016). Contrary associations were reported by Lacourpaille and others (Lacourpaille et al., 2017), who found increased SWV in elbow flexor and knee extensor muscles 30 min following high-repetition (>60) maximum voluntary eccentric contractions, as well as by Akagi et al., who reported an increase in triceps brachii stiffness immediately after a high effort (80% MVC) resistance training (Akagi et al., 2015). These contrary observations might be consistent insofar, in that muscle fatigue may lower while muscle damage may increase muscle stiffness (Ličen and Kozinc, 2022).

Muscular contraction likely modulates muscle shear properties through various factors. In a previous study, for instance, we found stretching of the tendon to result in an increase in SWV, which may be attributed to transient structural changes, such as collagen fibre relaxation and uncrimping, that occur after an initial load (Götschi et al., 2021; Purslow et al., 1998). Analogous mechanisms may be at play in the extracellular matrix of the muscle, in which collagen is a primary constituent (Csapo et al., 2020). Similarly, elevated perfusion leading to higher muscle blood volume that accompanies contraction may stretch the ECM, thereby increasing its apparent stiffness (Martin et al., 2018; Valic et al., 2005). The increased SWV could also more trivially be a result of involuntary low-level muscular activation following the high-effort contraction, although this mechanism is unlikely to be in effect over 5 minutes. Temperature changes in muscle tissue have been reported to be negatively associated with SWV (Bernabei et al., 2020). Related to its contractile component, preceding muscle activation may alter the myosin configuration caused by perturbations in intramuscular calcium homeostasis, thereby changing muscle shear properties (Colombini et al., 2010; Howell et al., 1993).

### The association of maximum eccentric hamstring strength with biceps femoris long head shear wave velocity

The relationship between a muscle’s elasticity and its capacity to perform physical work has been studied before (Akkoc et al., 2018; Saito et al., 2019; Yamazaki et al., 2022; Djurić et al., 2023).

Most relevantly, Saito et al. found muscle elasticity (rectus femoris and gastrocnemius, assessed with strain elastography) to be negatively associated with various measures of physical function (Saito et al., 2019). Of note, muscle volume was not indicative of these measures of physical function, which was also not the case in our study (data not shown). In our study, normalized MEHS was negatively associated with post-contraction SWV, although SWV at rest failed to reach statistical significance by a small margin and showed an analogous direction. The change in SWV in response to the exercise bout was, however, not indicative of the exerted force. It therefore appears conceivable that structural or compositional properties of the muscle that positively affect its capacity to produce force, negatively interact with the apparent shear modulus (as assessed by SWE) and that the preceding muscular contraction served as a preconditioning, attenuating confounding factors of the SWV.

Exploiting our novel 3D approach that enables the investigation of regional analysis, we also observed that this relationship predominantly exists in the central (radially) portion of the muscle, providing further ground for this latter conjecture. Muscular contraction increases the hydrostatic pressure in the muscle caused by muscle fibres being oriented non-parallel to the direction of net force production (Sejersted and Hargens, 1995) and by the Poisson effect, the phenomenon in which a material avoids volume change by expanding in directions perpendicular to the direction of compression, being opposed by the ECM (Wheatley et al., 2018). This tissue pressurization affects interstitial fluid distribution (Sleboda and Roberts, 2020; Fleckenstein et al., 1988) and impedes blood flow (Hill, 1948), and it increases from the periphery to the center of the muscle (Sejersted and Hargens, 1995). Consequently, the central muscle region likely experiences the largest perturbation of fluid distribution during contraction, possibly leading to an equalization of this confounder across participants.

The exact nature of the underlying factors simultaneously affecting eccentric muscle strength and SWV remains to be determined. Loss in muscle strength during aging or due to degeneration has been associated with an increase in connective tissue, in turn increasing muscle elasticity (Wen et al., 2018; Zaid and Goldspink, 1984). On an unexamined avenue, muscle strength is closely related to muscle fibre composition (Frontera et al., 2000), which may in turn influence SWV, potentially caused by a different extent of wave guidance due to the different diameters of the muscle fibre types (Frontera et al., 2008).

### Limitations

This study has limitations that should be addressed. Skeletal muscle is anisotropic, and rotations of the measurement plane (relating to the roll axis of the transducer) relative to the muscle fibre direction affect the measured SWV (Gennisson et al., 2010). Theoretically, not only probe orientation but also the load that is applied by the US transducer to the tissue may influence SWV (Gennisson et al., 2010; Eby et al., 2013), but this influence may be negligible at the loads to be expected during US examination (Alfuraih et al., 2018; Rominger et al., 2018). We tested this approach only in VL and BFlh and the reliability observed in this study could differ in other muscle groups. Furthermore, while performing repeated measurements of one subject in quick succession, as was done in this study, primarily provides information on the measurement reliability in terms of the technical aspects of the procedure, it may overlook potential unaccounted within-subject variability introduced by external factors. For instance, inter-day, as opposed to intra-day lower leg US elastography measurement repetitions, accounted for a drop of ∼0.15 in ICC in previous studies (Bravo-Sánchez et al., 2021; Taş et al., 2017). Of general note, whereas conversion of SWV into shear modulus is relatively simple in linearly elastic isotropic media, skeletal muscle may critically violate these assumptions, and we therefore decided to report SWV instead (Royer et al., 2011).

### Future perspectives

With the proof-of-concept provided here, future studies may apply analogous procedures to investigate research questions both in the realm of medicine, integrative muscle physiology, and sports science. For example, it is known that mechanotransduction is one of the main regulators of muscle growth and adaptations to exercise (Wackerhage et al., 2019). A previous study from our laboratory (Franchi et al., 2014) observed that distinct mechanotransduction proteins show region-specific activation after eccentric exercise only vs. concentric exercise only; notably, such responses were associated with changes in muscle morphology and architecture. As changes in mechanotransductor proteins (i.e., integrins) could be related to an increase in muscle stiffness (Csapo et al., 2020), our novel 3DSWE method could be used in combination with other physiological approaches in an integrative manner, in order to further describe and unravel the basic mechanisms of muscular adaptations to distinct exercise modalities.

One potential application is in the assessment of muscle function and performance. By providing quantitative spatially referenced measurements of muscle elasticity, the method could help evaluate the impact of training interventions, exercise protocols, and performance-enhancing techniques on muscle properties. This information could aid in optimizing training programs, monitoring muscle adaptations, and identifying potential areas of improvement or risk for injury. Additionally, the method can be valuable in understanding the biomechanics of specific sports movements and techniques by assessing the muscle properties involved. This can contribute to the development of evidence-based training strategies and injury prevention protocols tailored to the demands of different sports disciplines (Sarto et al., 2021).

## Conclusion

Three-dimensional mapping of skeletal muscle US shear properties as described herein provides reliable measurements and is capable of detecting variations both across anatomical locations and as induced by muscular contraction. A short high-effort exercise bout increases the SWV of skeletal muscle, the underlying mechanisms for which remain to be determined. Our finding that biceps femoris eccentric strength is associated with post-contraction SWV warrants further investigation.

## Data Availability

The original contributions presented in the study are included in the article/Supplementary material, further inquiries can be directed to the corresponding author.
